# Business and entrepreneurship is declining as scholarly field: Empirical evidence

**DOI:** 10.1371/journal.pone.0323297

**Published:** 2025-05-15

**Authors:** Wim Naudé

**Affiliations:** 1 RWTH Aachen University, Aachen, Germany; 2 University of Johannesburg, Johannesburg, South Africa; RMIT University, VIET NAM

## Abstract

This paper presents evidence from 68,792 papers published between 1961 and 2020 that progress in the scholarly field of business and entrepreneurship is declining. It is found that the annual number of papers published in the field has increased exponentially since the Second World War, growing on average by 17% annually since 1961; the average disruption score of papers have declined by a factor of 36 between the 1960s and the 2010s; and that the average team size per paper has increased from 1,6 between 1960–1980–2,4 between 2000 and 2020. Estimates from an ideas production function suggest that the field is getting fished out and that researchers may be stepping on one another’s toes. A Wald-test indicates that a structural break in the disruptiveness of business and entrepreneurship and papers occurred around 1999. These results could reflect pathologies in how research in the field is organized and/or that the field has matured.

## 1. Introduction

This paper presents evidence for the decline of business and entrepreneurship as research field based on the disruption index, team size and citations of 68,792 papers published in business and entrepreneurship between 1961 and 2020. This evidence suggests that the field has become a degenerating scholarly field marked by a) a rapid increase in the number of new papers published in the field per year, b) a decrease in the disruptiveness of publications in the field despite the increase in the number of publications, and c) research teams becoming larger over time.

While this evidence is tentative, mainly because of the current limitations of disruption indices it is nevertheless consistent with the diagnoses of [1,2]. [1] is concerned that it most research in the field now “fails to challenge our take-for-granted assumptions” [1, p.1] and [2] suggested that “an unplanned growth in literature can impede advancement”, warning that “the exponential growth of literature in small business and entrepreneurship research in recent years has made salient just such risk.” [2, p.1095].

If it has indeed become a mature field, business and entrepreneurship will be no exception, implying that field-specific factors are not the cause of the maturity - and degeneration in the sense of [3]. Recent studies, e.g., [4–9], documented declines in R&D productivity and the disruptiveness of new papers and patents across a broad range of fields. The present paper contributes to this research agenda by focusing on the field of entrepreneurship and business, which has so far not been a central concern in any of the studies cited.

The rest of the paper will proceed as follows. “Methodology” describes the methods subsequently used to test the hypothesis that the business and entrepreneurship field is degenerating, as measured by outputs, team sizes and papers becoming less disruptive over time. “Empirical findings” contains the empirical findings. “Discussion and conclusions” concludes.

## 2. Methodology

This paper uses descriptive and regression analyses to determine whether progress in business and entrepreneurship research is declining.

First, the average Disruption Score or Index (**D**, also referred to as ***D***_**1**_) and the share of disruptive papers annually from 1960 to 2018 for the field of entrepreneurship and business studies are calculated, and it is evaluated whether average disruption by papers in the field has been declining or increasing over time.

Second, a knowledge production function for the field is estimated using OLS and measuring new knowledge creation by the proxy of a paper’s disruptiveness. From this, estimates are obtained from which inferences can be made about whether the field is being fished out and whether research in the field is hampered by organizational pathologies.

The hypothesis is that the field is a degenerating field in the sense that new knowledge is getting harder to find. This would be reflected in a) a rapid increase in the number of new papers published in the field per year, b) a decrease in the disruptiveness of publications in the field accompanying the increase in the number of publications, and c) research teams becoming larger over time.

### 2.1 Data

Data on the Disruption Score (D), team size and citations of 68,792 papers published in the field of business and entrepreneurship studies between 1961 and 2020 are used. This data was obtained from [10] who makes data available for 19 million Microsoft Academic Graph (MAG) field-of-study (FOS) papers over the period 1830–2021 on *Harvard’s Dataverse* website (See https://doi.org/10.7910/DVN/JPWNNK). The broad field of “business,” which encompasses entrepreneurship, is a MAG “top-level field of study” with a level 0 label of 8 assigned to it [10]. The dataset provided by [10] is associated with a peer reviewed paper in *Nature*, and is maintained on the Harvard Dataverse, where it has been available since 2019. This provides a strong quality check on the dataset, which provides a rationale for using it in this paper: in short, it is one of the best datasets currently available with which to test the hypothesis that the disruptiveness of research in the business and entrepreneurship scholarly field has declined over time.

The disruptiveness of a paper is measured by the disruption score (D) (or *D*_1_), which has been calculated for each paper by [9] using citation network analysis, which is “based on the dynamic network measure of technological change introduced by Funk and Owen-Smith (2016)” [11, p.331].

[9, p. 378] describe their data sources as “ (1) the Web of Science (WOS) database that contains more than 42 million articles published between 1954 and 2014, and 611 million citations among them; (2) 5 million patents granted by the US Patent and Trademark Office from 1976 to 2014, and 65 million citations added by patent applicants; (3) 16 million software projects and 9 million forks to them on GitHub (2011–2014), a popular web platform that allows users to collaborate on the same code repository and ‘cite’ other repositories by copying and building on their code.”

From this data [9] define disruption (D) of a focal paper (FP) *i* with respect to papers that reference it (papers *j*) and papers that do not (papers *k*) by calculating “the difference between the proportion of type i and type *j* papers (*p*_*i*_ *− p*_*j*_) which equals the difference between the observed number of these papers (*n*_*i*_ *−n*_*j*_) divided by the number of subsequent works (*n*_*i*_ + *n*_*j*_ + *n*_*k*_)” [9], p. 389. Note that *n*_*k*_ “is the number of papers citing at least one of the FP’s cited references without citing FP itself” [12, p.1150]. Formally,


$$D=p_{i}-p_{j}=\frac{n_{i}-n_{j}}{n_{i}+n_{j}+n_{k}}$$ (1)

In other words, if an FP is cited as well as a significant number of its references, then the article is more consolidating of the field rather than disruptive [11]. The value of D ranges between 1 (most disruptive) to 0 (neutral) and -1 (least disruptive or “developing”).

According to [11, p.332] [9] “the disruption index does, in fact, measure what it intends to measure: new insights, ideas or methods that disrupt the cumulative nature in a scientific field.” Other studies that tested the validity of the disruption (D)-index include [13] and [14].

### 2.2 Regression model

To examine the hypothesis that the field of business and entrepreneurship has reached maturity and that it is a degenerating field, an ideas production function will be estimated based on data on Disruption Scores (D), team size and citations of 68,792 papers published in the field of business and entrepreneurship studies between 1961 and 2020, obtained from the data associated with [9] and made available by [10] via the website: https://dataverse.harvard.edu/dataset.xhtml.

**Fig 1:**
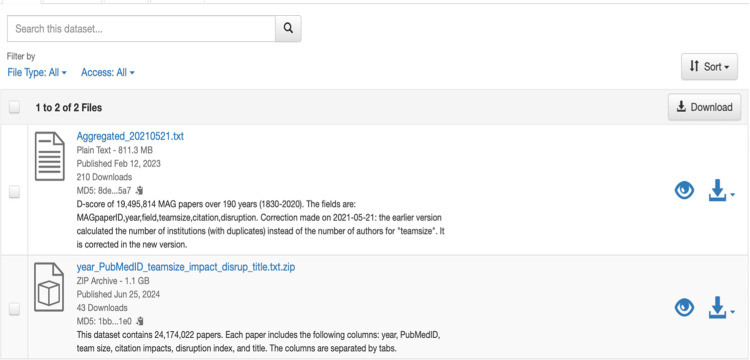
Access to the Full D-score Dataset of [9]. (Source: Screenshot from https://dataverse.harvard.edu/dataset.xhtml).

On this website, the authors provide detailed explanations of this dataset For present purposes, as noted, this paper only use the data pertaining to the field of business and entrepreneurship, field 8 in the data of [10]. Below, in “Descriptive results”, more details about this data are provided. For now, however, Fig 1 contains a screenshot of the access to the full data as it can be found on the mentioned website. Note that, although it would be useful to list the data as appendix to this paper, it is hardly feasible due to the size of the data, which spans detail om the disruption scores, team size and citations of 68,792 individual papers.

This data can be used in a standard ideas production function, which can be written as:


$$\Delta A_{t}=R^{\lambda }A_{t}^{\varphi }$$ (2)

According to (2), new knowledge in the field comes from the total number of entrepreneurship and business researchers/ scholars (*R*) working to discover new knowledge, as well as from the existing stock of knowledge, which inspires new ideas and can be recombined into new combination (see, e.g., [15]).

The sizes and signs parameters *λ* and *ϕ* are essential for the eventual growth of new knowledge (∆*A*). If *ϕ > *0 then intertemporal spillovers of knowledge are positive, and we have what has been termed the *standing-on-shoulders* effect, after Sir Isaac Newton who is reported to have said “If I have seen further, it is by standing on the shoulders of giants.” If, on the other hand, *ϕ < *1, there is what has been termed a *fishing-out* effect, after the case of a pond with only a limited number of fish, which diminishes over time. In such a case, just like it will be harder to catch new fish in a pond with dwindling supplies, if *ϕ < *1 then knowledge may be getting fished out.

As for the parameter *λ*, it measures the productivity of the researchers seeking new knowledge. If *λ > *1 there are complimentary effects between the efforts of the researchers – the more there are the better everyone will function. However, if *λ < *1, the productivity of a researcher will decline the more other researchers there are. It is a *stepping-on-toes* effect.

What do empirical evidence suggest about the signs and sizes of *ϕ* and *λ*?

There are no estimates so far for entrepreneurship and business studies—this paper provides the first tentative estimates. However, two sets of estimates have been provided for knowledge creation in the USA as a whole. The first, by [4] found *ϕ *= −2, 1. This means that ideas are getting fished out.

A second set of empirical estimates of *ϕ* and *λ* have been provided by [6] who found an estimate of *ϕ *= 1*,* 02–1,24. This means ideas are not getting fished out. However, they found that research productivity in the USA has been declining because of a significant stepping-on-toes effect. Their estimate of *λ* is -0.1, reflecting, as they put it, “*pathologies in how R&D is organized*.”

In sub-“Regression results”, the OLS regression results for estimating (2) in logarithmic form are reported.

## 3. Empirical findings

This section reports on the empirical findings on the disruptiveness of research in the broad business field, which encompasses research on entrepreneurship. “Descriptive results” presents descriptive statistics, and “Regression results” the regression analyses.

### 3.1 Descriptive results

The summary statistics of the variables obtained from [10] are contained in Table 1. Table 1 shows that across the 68,792 papers of interest, the average paper had around 43 citations and 2,3 authors, and its disruption score was 0,012. The largest team size was 49 authors per paper (a 2020 paper), and whereas the average citations per paper was 43, the most citations were 29,538 (for a paper published in 1981).

**Table 1:** Summary statistics, 68,792 papers in entrepreneurship and business studies, 1961–2020.

Variable	Mean	Standard Deviation
Disruption	0,012	0,074
Team size per paper	2,3	1,30
Citations per paper	42,7	208,4

Source: Authors’ own compilation.

Finer analysis of this data allows one to document the growth and development of the field, including the disruptiveness of research and the sizes of research teams over time. First, as far as the growth and development of entrepreneurship as a scientific field are concerned, the field emerged in the 1960s and accelerated with exponential growth in publications in the 1990s - the average growth rate of new papers has average 17% since 1961.

This exponential rise in publication output is a feature that entrepreneurship as a field shares with many other fields. While it reflects success in mobilizing resources and getting results, [5] have warned that it may reflected a bias in favour of quantity rather than quality. This is the case in entrepreneurship research, too, as [2] has warned.

Second, as far as the disruptiveness of papers in the field is concerned, the data of [10] consistently point out that the disruptiveness of papers in the field has declined in spite of the rapid growth in publications (and the increase in research team size). The D- score value has declined from an average of 0,183 in the 1960s, to 0,005 in the 2010s. This is a 36-fold decrease.

**Fig 2:**
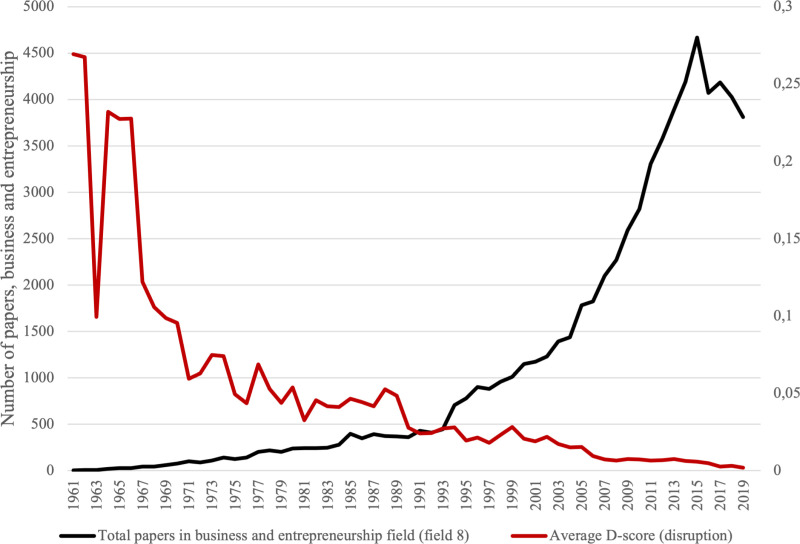
Disruption scores and total papers, 1961–2019. Source: Author’s compilation based on data from [10].

Fig 2 depicts the decline in disruptive papers as measured by the D-score proposed [9] against the increase in average team size.

**Fig 3:**
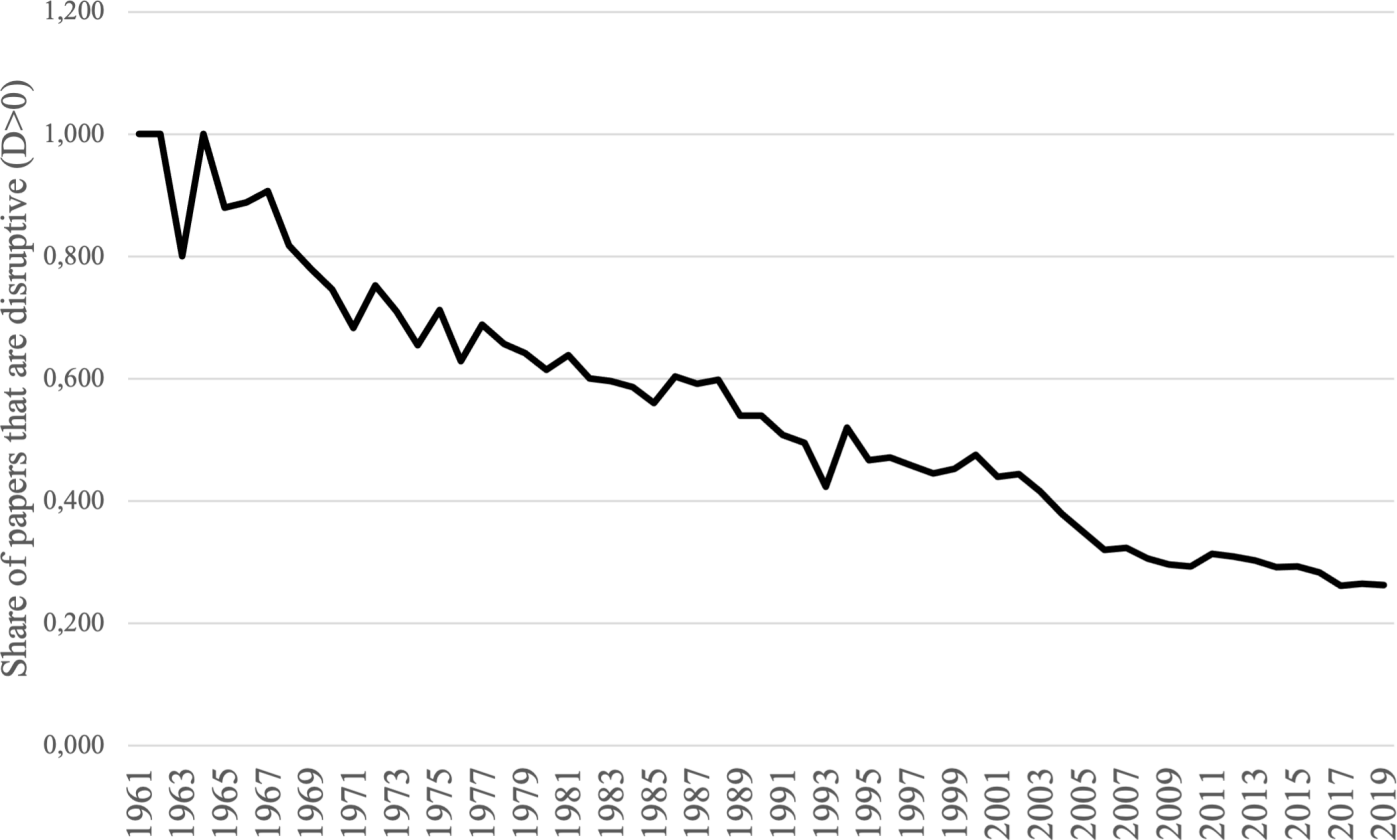
Share of Papers with a D > 0, 1961–2019. Source: Author’s compilation based on data from [9].

One can also calculate the share of papers annually that had a D-score *>* 0, i.e., which were somewhat disruptive. Fig 3 shows that, as with the average D-score, the share of papers with a *D >* 0 has consistently declined over time.

Third, regarding the size of research teams per paper, the data shows that the average team size involved in a paper increased over time. Table 4 below shows that the average team size per paper increased from 1,6 between 1960–1980–2,4 between 2000 and 2020.

**Table 4:** Summary Statistics for Start and End of the Sample Period.

Variable	Mean, 1961–1980	Mean, 2000–2020
Disruption	0,070	0,0070
Team size per paper	1,59	2,42
Citations per paper	62,95	29,4
N	1865	57164

Source: Authors’ own compilation.

Around 27% of all papers published between 1961 and 2020 were solo-authored papers, which is very similar to the share of 24% found by [9] in a sample of 43,661,387 papers across all fields from 1900 to 2014. Notably, between the 1960s and the 2010, the share of all papers by solo authors declined from 68% to 19%. The finding here of growth in team size over time in the publication of papers in the entrepreneurship and business field is consistent with findings from other fields, such as those of [9,16,17].

The simultaneous decline in the disruptiveness of papers and the increase in research team sizes over time in the field of entrepreneurship and business, is consistent with the dynamics of the rise and fall of scientific fields as documented by [8]. The latter, using 1.5 million articles on ArXiv covering 175 research fields found that “the early phase of a field is characterized by disruptive works mixing of cognitively distant fields written by small teams of interdisciplinary authors, while late phases exhibit the role of specialized, large teams building on the previous works in the field” [8, p.1]. This seems to be an apt characterization of the data patterns reported in this section.

### 3.2 Regression results

Using OLS, a standard ideas knowledge production function (equation 2) is estimated in log format, i.e.,


$$\Delta \log A_{t}=a_{0}+\lambda logR+\phi logA$$ (3)

Where ∆*logA* is the change in the log of the total disruption score in a year; *logR* the log of the number of researchers publishing in the field in a year, and *logA* the log of the cumulative knowledge in the field as proxied by the cumulative D-score. The assumption here is that the D-score reflects the production of new knowledge, which is not unreasonable as the D-score measures the extent to which a paper is “novel” in the sense of not citing as much of the standard references.

The regression results are contained in Table 2. The overall results are highly significant, with a *R*^2^ of 0,74. All the parameters are statistically significant at the 1% and 5% levels.

**Table 2:** OLS regression results, ideas production function for the field of entrepreneurship and business, 1961–2018, dependent variable ∆A.

Variable	Coefficient	t-value
constant	0,61	5,80***
logR	0,11	2,60**
logA	-0,23	-3,33***
*Diagnostics:*		

*R*^2^ = 0,74.

F (2,53) = 26,77*** N = 56.

* and ** indicates significance at the 1% level and ** at the 5% level (calculated using robust standard errors).

The *Supremum Wald test* (see [18]) for an unknown structural break in the time series was performed following the regression. The null hypothesis of no structural break was rejected (test statistic value was 20,06 with an associated p-value of 0,003), and the break date was estimated as 1999.

The sizes and signs on the *ϕ* and *λ* are respectively -0,23 and 0,11. Given that *ϕ < *1 there is a fishing-out effect. Also, given that *λ < *1 the implication is that the impact of a researcher in entrepreneurship and business is declining given the more other researchers there are. It is a stepping-on-toes effect. That the fishing out of ideas are driving team size increases is confirmed by the result that if the total number of researchers publishing in the field in a particular year is substituted with the average size of the teams that produce a single paper, then further support for the fishing out of ideas is found- see Table 3.

**Table 3:** OLS regression results, ideas production function for the field of entrepreneurship and business, 1961–2018, dependent variable ∆A, with the average size of publication teams.

Variable	Coefficient	t-value
constant	-1,58	-1,41
logR	0,32	1,78**
logA	-0,13	-3,29***
*Diagnostics:*		

*R*^2^ = 0, 69.

F (2,53) = 19,10*** N = 56.

** indicates significance at the 1% level and ** at the 5% level (calculated using robust standard errors).

The results in Table 3 suggest that, controlling for fishing-out effects, larger team sizes are driving disruptive papers. With *λ < *1, this is not, however, leading to increasing returns but rather decreasing returns, confirming a stepping-on-toes effect.

To further investigate how the nature of teams and disruptions in the field of business and entrepreneurship had changed between the 1960s-1970s and 2000–2020, the sample was split into these two bookend periods, the summary statistics for each calculated, and a regression performed of the D-score on the team size for both periods. This enables one to compare the disruptiveness of papers at the beginning and the end of the period and examine how the relationship between team size and disruption has changed over time.

The summary statistics for the two periods are contained in Table 4.

Table 4 shows that papers in the later period (2000–2020) were on average 10 times less disruptive than in the later period (2000–2020), had only 46% of the citations of earlier papers, and required on average a team of authors that were 52% larger.

Table 5’s results confirm a negative relationship between team size and the disruptive nature of papers - smaller teams produce more disruptive papers. However, the negative relationship between team size and disruption has become smaller over time. This suggests that although small teams produce more disruptive papers in entrepreneurship and business, these smaller teams have become less effective over time.

**Table 5:** OLS regression results, dependent variable D-scores.

Variable	Coefficient (1961–1980)	Coefficient (2000–2020)
constant	0,10 (10,26)***	0,01 (24,81)***
Team size per paper	-0,02 (-3,47)***	-0,00 (-11,68)***
adj *R*^2^	0,01	0,00
N	1865	57164

Source: Authors’ own compilation.

## 4. Discussion and conclusions

This paper used data on disruption scores, team size, and citations of 68,792 papers published in business and entrepreneurship studies between 1961 and 2020 to conclude that there is a decline in the disruptiveness of papers on the business and entrepreneurship scholarly field. This may reflect that the field is losing its luster. Several scholars have been suggesting that not all is well in the field. It can for instance be observed that entrepreneurship research is increasingly going around in circles, revisiting topics dealt with in the past, often only introducing new labels and casting old wine in new bottles. [19, p.275] expressed concern that the “vitality of the academic field of entrepreneurship is not at all guaranteed” because it may be in danger of losing touch “with the real-world phenomenon it is trying to explain and understand. [2, p.1096] are alarmed by more and more publications with “trivial findings” and “little novelty.” [20] argues that the field of entrepreneurship is obsessed with economic and firm growth which constrains it relevance for real-world phenomenon, such as climate change and ecological overshoot.

The question is, does this the finding in this paper that the D-score of business and entrepreneurship research is declining represent a regrettable degeneration of the scholarly field of business and entrepreneurship, or are there other explanations?

One explanation could be that the D-score used to measure disruption of papers may over-estimate the extent of decline. While it has been noted in “Data” that the disruption index have been argued to measure what it intends to measure [11] and that the validity of the disruption (D)-index have been confirmed in studies [13,14], it is nevertheless the case that the measure also has limitations. Discussions of the strengths and limitations of the Disruption (D) score of [19] - and an overview of its uses - are contained [21] and [22]. The latter point out that the most significant weakness of the D-score is that it “detects only a few papers as disruptive due to the term *N*_*k*_, which is often very large compared to the other terms in the formula [...] A large *N*_*k*_ produces disruption values of small magnitude, as *N*_*k*_ only occurs in the denominator of the formula. As a result, the disruption index is very similar for many papers, and only a few papers get high disruption values” [22, p.1245].

Hence, “More research on the validity of disruption scores as well as a more precise understanding of disruption as a theoretical construct is needed before the indices can be used in the research evaluation practice” [21, p.1]. Furthermore, research is needed to estimate the ideas production function for the field using better and diverse measures of knowledge creation.

A second possible explanation for the results could be, not that business and entrepreneurship scholarship is declining or degenerating in a negative sense, but that the field has matured. In other words, scholars in business and entrepreneurship have been successful in solving the field’s scientific challenges. This finding is consistent with the results of a recent literature survey by [1, p.1] who concluded that *“Entrepreneurship has matured as a research field.”*

It could be countered that, given that the field is a relatively young field, that such maturation is pre-mature. This possibility - of premature maturation - would require further, for instance to understand better how teamwork has evolved in the field, and whether different organization of research teams, for example, in terms of hierarchy, results in more or less disruptive papers.

Finally, if the field of business and entrepreneurship is indeed facing diminishing returns to research, which the tentative findings in this paper suggest, it may imply, appropriately for a field studying entrepreneurship, an opportunity since, as [23] advises, “*Instead of rejecting the concept of ending, scholars across disciplines should use this moment to ask: why do we do what we do, and when (if ever) could we be done?* ” [20,24] have suggested that business and entrepreneurship studies have been “obsessed” with firm growth and business growth, and that given the link between these and ecological overshoot, there is a need for business and entrepreneurship scholarship to engage with the need for a post-growth society. Moving beyond the growth obsession and reframing the end-goal of business and entrepreneurship away from being a tool to growth, may reinvigorate the field to the extent that it may be in decline and stagnating.
